# The KRESCENT 2.0 Health Research Training Platform Application Process: Program Report

**DOI:** 10.1177/20543581251364309

**Published:** 2025-08-21

**Authors:** Veronica Kaye, Tarrah Wood, Jennifer Klein, Leanne Stalker, R. Todd Alexander, Adeera Levin, Sunny Hartwig

**Affiliations:** 1 Department of Biomedical Sciences, Atlantic Veterinary College, University of Prince Edward Island, Charlottetown, Canada; 2 Division of Pediatric Nephrology, Department of Pediatrics, University of Alberta, Edmonton, Canada; 3 The Kidney Foundation of Canada, Montreal, QC, Canada; 4 Department of Internal Medicine, The University of British Columbia, Vancouver, Canada

**Keywords:** Kidney Research Scientist Core Education National Training Program (KRESCENT), research capacity building, CIHR HRTP grant application process, Patient Community Advisory Network (PCAN), Equity, Diversity and Inclusion (EDI) principles

## Abstract

**Purpose of Program:**

The Kidney Research Scientist Core Education and National Training Program (KRESCENT) was launched in 2005 to enhance kidney research capacity in Canada and foster knowledge translation across the 4 pillars of health research. This program report describes the pan-Canadian KRESCENT 2.0 Health Research Training Platform (HRTP) application process that was awarded a 5-year grant through the pilot Canadian Institutes of Health Research (CIHR) HRTP program, ensuring continuation of this capacity-building program in Canada.

**Sources of Information:**

Grant application documents including meeting minutes, break out group summaries and recommendations, and Gantt timeline charts. Other resources included websites and journal articles.

**Methods:**

All application-related documents were reviewed. Clarification of process and timelines was provided through interviews with the Nominated Principal Applicant (NPA) Dr R. Todd Alexander, Principal Applicants (PAs) Drs Adeera Levin and Sunny Hartwig, Project Manager (PM) Dr Jenn Klein, members of the Patient Community Advisory Network (PCAN), and the Kidney Foundation of Canada Program (KFoC) Manager Ms. Julie Wysocki via in-person and virtual meetings as well as email correspondence.

**Key Findings:**

The KRESCENT 2.0 HRTP application represents a 6-month pan-Canadian effort spearheaded by the NPA and a pan-Canadian team of PAs spanning multiple jurisdictions, disciplines, and sectors. Early engagement of stakeholders in the Canadian kidney research community, outstanding PM administrative support from the onset of the application process were identified as pivotal for the success of the application. Other essential factors for success included graphic design assistance to effectively communicate key and complex concepts, appointment of an EDI champion, engagement with a diverse group of collaborators, and strategic collaboration with other HRTP grant applicants to navigate the ambiguities of the pilot HRTP call. Indispensable, scrupulous final review of the complete application package was generously provided by Dr Robert Quinn (University of Alberta) prior to final grant submission to CIHR.

**Limitations:**

Unlike other funded HRTP applicants, KRESCENT is an established kidney training platform for a small cohort of trainees. Our results may not generalize well to HRTPs with large group cohorts or newly established HRTPs.

**Implications:**

This program report may provide valuable guidance for other groups seeking to successfully navigate the CIHR HRTP application process.

## Introduction

Chronic kidney disease (CKD) is a global public health challenge, affecting 4 million Canadians and 1 in 10 people worldwide,^1-3^ with a staggering economic burden of more than $40 billion per year in Canada.^2^ KRESCENT originated with recognition by the Canadian research community of the rising prevalence of kidney disease, coupled with declining interest and engagement of trainees in kidney research.^4-7^ At its outset, the major objectives of the program were to enhance kidney research capacity in Canada by training the next generation of leading investigators and to improve collaborations and knowledge translation (KT) across the four themes of health research: biomedical, clinical, health systems and services, and social, cultural, and environmental factors that affect the health of populations.

Initial funding was jointly provided by CIHR, KFoC, and the Canadian Society of Nephrology (CSN). A steady decline in the Canadian health research funding in subsequent years has presented a significant challenge to long-term sustainability of KRESCENT and other training programs. In 2021, CIHR launched the HRTP pilot grant program with the express mandate to:

*fund health research training platforms in the development of interdisciplinary, interjurisdictional, and intersectoral research to attract and retain a diverse array of outstanding trainees and early career researchers (ECRs) and provide the necessary skills for success in both academia and industry*^8^ (https://cihr-irsc.gc.ca/e/53595.html)

HRTP applicants were required to meet the following objectives to be eligible for funding:

1. Support comprehensive training by engaging academic, non-academic, and knowledge user mentors from across a variety of disciplines, sectors, and jurisdictions.
2. Promote a greater understanding of emerging research and knowledge exchange approaches.
3. Increase training and professional development in support of sustainable career trajectories.
4. Consider best practices in EDI within the platform team and environment.
5. Demonstrate a value-add and provide training and mentoring opportunities which go above and beyond standard research training programs that trainees typically experience.

Twelve grants were available, with two grants in the “Nutrition, Metabolism, and Diabetes” pool totaling $2.4 million each, with $400 000 payable annually per year for 6 years. Here, we describe the KRESCENT 2.0 HRTP grant application process (Table 1). Founded upon the original KRESCENT curriculum (ie, KRESCENT 1.0) and faithful to its mission and values,^4,5^ the KRESCENT 2.0 curriculum nevertheless incorporates multiple substantive changes that were necessitated by the HRTP application eligibility requirements.

**Table 1.** HRTP Grant Application Process Milestones and Timelines.

Event	Date and timing (2021)
HRTP pilot grant call for applications	T-minus 7 months
Strategic planning meetings (KRESCENT team)	T-minus 7 months
Hiring of PM and graphic designer	T-minus 7 months
Establishment of project governance and identification of participants (NPA, PA, co-applicants, collaborators)	T-minus 6.5 months
Initial small group meeting (16 participants)	T-minus 6 months
Appointment of EDI champion	T-minus 6 months
PCAN consultation	T-minus 6 months
Pan-Canadian (large group) meeting (42 participants)	T-minus 5.5 months
Pan-Canadian (small group) working group meeting (24 participants)	T-minus 5 months
Submit CVs (NPA and PAs) and grant registration	T-minus 4.5 months
KRESCENT industry meeting	T-minus 4.5 months
Registration deadline	T-minus 4 months
Submission of a full CIHR academic CV Collation and uploading of common CVs from remaining participants into CIHR application portal	T-minus 4 months
Internal review of draft grant application (KRESCENT and KFoC) Completion of budget (performed by NPA and KFoC) and establishment of a host institution (affiliated with the NPA) to administer funds	T-minus 3.5 months
External review of draft grant application (Dr Robert Quinn, University of Alberta)	T-minus 2.5 months
External review (funded by WCHRI) supplied and provided funding for the external editor (June 14, 2021).^a^	T-minus 2.5 months
Submission of application (application deadline)	T = 0
Funding start date	January 2022
Notice of decision^b^	February 25, 2022

Abbreviations: CIHR, Canadian Institutes of Health Research; CV, curriculum vitae; HRTP, Health Research Training Platform; KFoC, Kidney Foundation of Canada; KRESCENT, Kidney Research Scientist Core Education and National Training program; NPA, nominated principal applicant; PA, principal applicant, PCAN, Patient Community Advisory Network; PM; project manager; POR, patient-oriented research; WCHRI, Women and Children’s Health Research Institute.

a Funding for the 0.5 FTE project manager and for external review during the application process was provided by WCHRI at the University of Alberta, in accordance with its mandate to support initiatives to increase large grant funding to NPAs of the Institute.

b The start of funding preceded the official notice of decision.

## Sources of Information and Methods

Grant application documents comprised the bulk of sources of information for this program report, including meeting minutes, break out group summaries and recommendations, and Gantt timeline charts. Other resources included websites and journal articles. Both during and after the review of all application-related documents, clarification of process and timelines was provided through interviews with the NPA, PAs, PM, PCAN members, and the KFoC Program Manager, via in-person and virtual meetings as well as email correspondence.

## Key Findings

### Establishing Project Management

Given its infancy, it is perhaps unsurprising that the HRTP application instructions and requirements were challenging to interpret. The application document was lengthy (28 pages); expectations and instructions were distributed throughout the document and in places were vaguely and/or ambiguously stated. Contradictory instructions could be found in different sections of the grant application. The PM was therefore instrumental in helping to decipher and interpret the grant submission guidelines and instructions. Given the complex organizational logistics of managing a large pan-Canadian team across multiple time-zones, the PM also provided indispensable administrative support in managing deadlines (Table 1), collecting and formatting CVs to CIHR format, organizing virtual meetings and timelines, iterative upkeep of a Gantt chart (a tracking tool that visually monitors progress of grant application tasks; Supplementary Table 1), and highlighting important information gaps throughout the application process. The PM was also instrumental in liaising with CIHR throughout the application process to clarify grant parameters. Funding for the 0.5 FTE PM position was obtained through WCHRI—affiliated with the NPA.

In addition to the selection of the PM, graphic design assistance provided critical visual conceptual aid. Collaborative discussions with other HRTP PAs, namely, Empowering Next Generation Researchers in Perinatal and Child Health (ENRICH) and Training Researchers in the Next Generation in Gastroenterology and Liver (TRIANGLE) provided important strategical guidance in clarifying ambiguity of language and expectations of the pilot application process. Roundtable discussions with stakeholders were held throughout the HRTP application process as needed.

### Early Engagement With Stakeholders in the Canadian Kidney Research Community Established the Priority Areas and Actionable Items for the KRESCENT 2.0 Application

Six months prior to the application deadline (T-minus 6 months), an initial virtual strategic planning meeting was held with 16 stakeholders in the Canadian kidney research landscape. This first planning meeting was a crucial first step in charting the course for the KRESCENT 2.0 application process. By unanimous consensus, several priority areas were identified:

1. Increased training in sex/gender.
2. Increased training of Black and Indigenous kidney health researchers and increased focus on research in kidney health and disease of Black and Indigenous communities.
3. Greater incorporation of Patient-Oriented Research (POR) across the pillars.
4. Exposure to novel techniques that can be used to understand kidney health and disease (ie, machine learning, omics, pragmatic randomized control trials, n-of-1 trials, etc).
5. Identification of other priority areas required for grant success.

Subsequently, co-applicants with corresponding expertise were proposed during this meeting (please see Supplementary Table 2 for the final grant participant matrix table).

**Figure 1.**
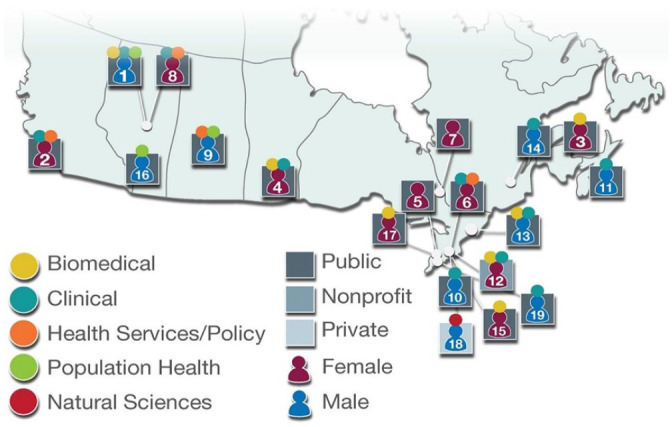
The KRESCENT mentorship team spans across jurisdictions, sectors, and disciplines. 1. Todd Alexander (NPA). KRESCENT Program Director. 2. Adeera Levin (PA), KRESCENT leadership since inception. 3. Sunny Hartwig (PA), KRESCENT Curriculum Chair and KRESCENT alumna. 4. Julie Ho (PA), KRESCENT alumna. 5. Leanne Stalker (PA), Scientific Director of KFoC. 6. Janine Farragher (PA), ECR and KRESCENT alumna. 7. Mary Beaucage (PA), Patient Council member. 8. Kara Schick-Makaroff (PA), KRESCENT alumna. 9. Malcolm King (PA) Indigenous Scholar and former head of the CIHR Institute of Aboriginal Health. 10. Sanjay Pandeya (PA), President of the CSN. 11. Karthik Tennankore (PA). 12. Ana Konvalinka (PA), KRESCENT alumna. 13. Kevin Burns (PA), KRESCENT Founder and former Director. 14. François Madore (PA). 15. Nina Jones (Co-App), KRESCENT alumna. 16. Robert Quinn (Co-App). 17. Lisa Robinson (Co-App) EDI Champion. 18. Morteza Ahmadi (Co-App), KRESCENT alumni entrepreneur. 19. Mitch Abrams (Co-App). Abbreviations: CIHR, Canadian Institutes of Health Research; Co-App, co-applicant; ECR, early career researcher; KFoC, Kidney Foundation of Canada; KRESCENT, Kidney Research Scientist Core Education and National Training program; NPA, nominated principal applicant; PA, principal applicant.

Next (T-minus 5.5 months), a large and diverse meeting was held of stakeholders across multiple sectors and jurisdictions (Figure 1) in the Canadian kidney research community (n = 42) in order to firmly establish the priorities for the KRESCENT 2.0 grant application. Following an initial discussion of the grant application call, participants were randomly assigned to small breakout groups, each tasked with establishing definitive priorities for the KRESCENT 2.0 application. Participants then reconvened as a large group and together, by unanimous group consensus, established 6 concrete priority areas for the KRESCENT 2.0 application:

1. Opportunities for Black and Indigenous scholars.
2. Sex and gender.
3. Exposure to novel techniques.
4. POR.
5. Mental health.
6. Industry partnerships.

In the remaining time, participants returned to their same breakout rooms, tasked with ways to actualize the first three priorities into specific action items for the KRESCENT 2.0 application. Two weeks later (T-minus 5 months), the remaining three priorities were discussed among a smaller subset (n = 24) of the large group participants. Following essentially the same format as the previous meeting, participants were first assigned to breakout groups to encourage robust discussions in a small group setting and then reconvened to discuss with the entire group. Priority areas and actionable items for the KRESCENT 2.0 grant application are summarized in Table 2.

**Table 2.** Priority Areas and Actionable Items for the KRESCENT 2.0 Grant Application.

Priority area	General description	Actionable item(s)
1. Opportunities for Black and Indigenous scholars	Increase number of Canadian Black and Indigenous kidney researchers Increased support for research that supports the kidney health and care of Black and Indigenous communities	Establish a targeted KRESCENT fellowship to support a trainee doing research supporting kidney health and care in Black and/or Indigenous communities in Canada Launch a summer studentship program to support kidney research opportunities for black and Indigenous students
2. Sex and gender	Enhanced training opportunities within curriculum Addressing gender equity Going beyond traditional sex and gender; incorporation of non-binary Opportunities to link with interest groups and content experts	Additional mandatory online modules for sex and gender training In-person didactic lectures on sex and gender training incorporated into KRESCENT core curriculum Establishing a dedicated KRESCENT fellowship seat for sex and gender kidney research Highlighting online training modules available on the ENRICH platform (e.g., Women in Transplantation)
3. Exposure to novel techniques	Incorporate didactic training in novel techniques into the curriculum Involve experts in unique novel techniques to participate in workshops and online modules^a^ Add elective component into curriculum for specialized techniques Provide funding for trainees to travel and learn new techniques	In-person didactic lectures with a focus on cutting edge techniques All invited speakers are tasked with highlighting innovations in their field as part of their lecture Highlighting online training modules available on the ENRICH platform
4. POR	Integrate patients into research programs Deeper integration of the lived experience voice into the curriculum Create a patient/mentor program for trainees Establish novel relationships with hospitals and volunteers	Formalization of the KRESCENT PCAN team Increasing participation of PCAN beyond patient stories at workshops toward practical mentorship in improved lay communication (mentorship in writing lay abstracts led by PCAN, and ongoing offer to provide one-on-one mentorship for trainees) PCAN members participate in the transdisciplinary research challenge exercise with trainees and share co-authorship on the resulting manuscript
5. Mental health	Increased emphasis on trainee well-being	Greater investment of health and wellness modules in KRESCENT workshops (eg, nature as medicine, forest bathing, mindfulness, group outings to art galleries, and didactic lectures) Invited speakers tasked with sharing ways they have navigated overall well-being during their career Highlighting online training modules available on the ENRICH platform and other resources (eg KidneyLink)
6. Industry partnerships	Greater exposure to non-academic career pathways	Guidance on alternate career pathways incorporated into KRESCENT curriculum Helping trainees connect with mentors within industry Connect with KRESCENT alumni who chose a non-academic pathway

Abbreviations: ENRICH, Empowering Next Generation Researchers In Perinatal and Child Health; NPA = nominated principal applicant; PA = principal applicant; PCAN = patient Community Advisory Network; POR, Patient-oriented research.

a Prioritization of inviting Canadian content experts (vs international speakers) to participate in KRESCENT workshops provides important networking opportunities for trainees with senior Canadian researchers in a small group setting.

**Figure 2.**
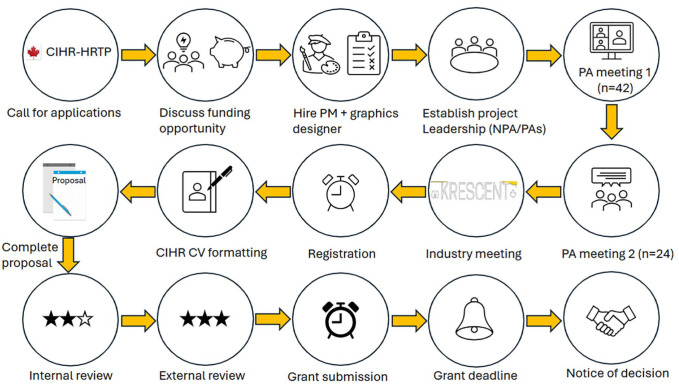
The HRTP application process.

Spearheaded by the NPA, the 232-page KRESCENT 2.0 grant application package was constructed over a period of several months, undergoing extensive internal and external review prior to its submission to the CIHR Institute of Nutrition, Metabolism and Diabetes (INMD) 1 month ahead of deadline. The HRTP application process is summarized in Figure 2.

### Unique Budgetary Considerations and Challenges of the KRESCENT 2.0 Application

Historically, KRESCENT funding has been provided by CSN, KFoC, CIHR-IMND, industry partners, and matching funding from trainee institutions. Support from KFoC represents targeted fund-raising initiatives to partners and sponsors including provincial agencies, private industry and individual donors. In addition, indispensable “in-kind” administrative support has been provided by KFoC since program inception. KFoC administrative support for KRESCENT was formalized into the HRTP application budget as a full-time program manager-level role. This guarantee of upper managerial administrative support by KFoC was a key budgetary strength of the KRESCENT application, directing all potential HRTP funds toward supporting the program itself, rather than its administration.

Creation of the budget to accompany the HRTP application for the KRESCENT program was painstaking and complex. Given its ongoing funding, the HRTP budget had to account for, integrate, and balance pre-existing funding to KRESCENT with new CIHR funds being requested. The budget for the KRESCENT 2.0 curriculum was also tasked with balancing the inclusion of novel curriculum elements while maintaining ongoing program aspects, to ensure the maintenance of existing key program elements while allowing program expansion into the important areas dictated by the grant initiative (eg, EDI). In addition, the budget was required to account for substantially increased operational costs post COVID, as well as increased program management requirements that accompanied program expansion. Notably, CIHR funding from the KRESCENT program had been withdrawn 1 year prior to the HRTP application. KFoC generously increased their financial contribution in order to continue to fund as many trainees as possible. Some offset of cost was provided by COVID-era program savings.

Added complexity came with distinct categorization of spending outlined in the HRTP requirements. All proposed budget elements were required to adhere to existing CIHR spending specifications and limits (eg, no more than 60% of funds could be used to support trainee stipends). It was therefore necessary to utilize external stakeholder funds to balance categorized spending where program requirements exceeded CIHR limits.

Re-wiring of the funding pipeline was a key complexity of the KRESCENT budget design. Historically, program support flowed directly through KFoC, save CIHR salary support sent directly to individually supported trainees. To accommodate HRTP funds, a new financial administration portal was created at the grant host institution. Individual budget lines for the grant often had to be split between HRTP funds and KFoC partnership money in order to remain within CIHR guidelines. This proved exceedingly challenging, because of existing differences in financial guidelines between CIHR, the host institution itself, and KFoC.

In short, budget development for the HRTP was an extremely complex process. Many of the complexities presented within the budgeting process may have been unique to KRESCENT, in that its historic hosting within a charitable organization differs from most academic networks or training programs which are classically held at academic institutions through individual academics.

## Limitations

KRESCENT is an established kidney training platform for a small cohort of trainees. Our results may not generalize to large group cohorts other new or pre-existing HRTP training platforms.

## Implications

This process report describes the KRESCENT 2.0 HRTP pilot grant application, representing a 6-month pan-Canadian effort spearheaded by the NPA. Several elements were essential for the successful execution and subsequent funding of the KRESCENT 2.0 grant application: early engagement with partners in the Canadian kidney research community, outstanding project management and administrative support, graphic design assistance in providing simple and clear visual conceptualization of the pan-Canadian team, governance, and format of KRESCENT for the grant application, strategic collaboration with other HRTP applicants to interpret the details of the pilot CIHR application call, and scrupulous and careful final peer review prior to submission. It is hoped that this program report outlining the details of the lengthy and challenging HRTP application process will be of help to other groups seeking to establish or continue their training programs by obtaining funding through CIHR or other funding agencies.

## Supplemental Material

sj-docx-1-cjk-10.1177_20543581251364309 – Supplemental material for The KRESCENT 2.0 Health Research Training Platform Application Process: Program Report Supplemental material, sj-docx-1-cjk-10.1177_20543581251364309 for The KRESCENT 2.0 Health Research Training Platform Application Process: Program Report by Veronica Kaye, Tarrah Wood, Jennifer Klein, Leanne Stalker, R. Todd Alexander, Adeera Levin and Sunny Hartwig in Canadian Journal of Kidney Health and Disease

sj-docx-2-cjk-10.1177_20543581251364309 – Supplemental material for The KRESCENT 2.0 Health Research Training Platform Application Process: Program Report Supplemental material, sj-docx-2-cjk-10.1177_20543581251364309 for The KRESCENT 2.0 Health Research Training Platform Application Process: Program Report by Veronica Kaye, Tarrah Wood, Jennifer Klein, Leanne Stalker, R. Todd Alexander, Adeera Levin and Sunny Hartwig in Canadian Journal of Kidney Health and Disease
