# Supplementary material for: The KRESCENT 2.0 Health Research Training Platform Application Process: Program Report
Source: Can J Kidney Health Dis. 2025 Aug 21;12:20543581251364309. doi: 10.1177/20543581251364309 (PMC12374036; doi:10.1177/20543581251364309)
Supplement: sj-docx-1-cjk-10.1177_20543581251364309 – Supplemental material for The KRESCENT 2.0 Health Research Training Platform Application Process: Program Report [file sj-docx-1-cjk-10.1177_20543581251364309.docx]

Supplementary Table 1.

| **Name** | **Role(s)** | **Classification(s)** | **Sector*** | **Discipline**** | **Primary Institution/ Organization** | [**U15 Group of Canadian Research Universities?**](http://u15.ca/our-members) | **Province/ Territory** | **Part of PAC?** |
| --- | --- | --- | --- | --- | --- | --- | --- | --- |
| Alexander, Todd | NPA/Mentor | IR | P | Biomed, Clinical | U of A | Yes | AB | No |
| Levin, Adeera | PA/Mentor | KU, IR | P | Clinical | UBC | Yes | BC | No |
| Hartwig, Sunny | PA/Mentor | IR | P | Biomed | UPEI | No | PE | No |
| Ho, Julie | PA/Mentor | IR | P | Biomed, Clinical | UofM | Yes | MN | No |
| Stalker, Leanne | PA/Mentor/Platform Manager | KU | NP | Health Systems | UofGuelph | No | QC | No |
| Farragher, Janine | PA/Mentor | ECR | P | Health Systems | U of T | Yes | ON | No |
| Beaucage, Mary | PA/Mentor/PAdC | KU | P | Cultural | N/A | N/A | ON | No |
| Schick-Makaroff, Kara | PA/Mentor | IR | P | Health Systems | U of A | Yes | AB | No |
| King, Malcolm | PA/Mentor | IR | P | Cultural | U Sask | Yes | SK | No |
| Pandeya, Sanjay | PA/Mentor | KU | NP | Clinical | CSN | No | ON | No |
| Tennankore, Karthik | PA/Mentor | IR | P | Clinical | DAL | Yes | NS | No |
| Kovolinka, Ana | PA/Mentor | ECR | P | Biomed | U of T | Yes | ON | No |
| Burns, Kevin | PA/Mentor | IR | P | Basic Science | Ottawa U | Yes | ON | No |
| Madore, François | PA/Mentor | KU | P | Clinical | Montreal | Yes | QC | No |
| Robinson, Lisa | EDI/Co-App/PAC | IR | P | Biomed | U of T | Yes | ON | Yes |
| Quinn, Robb | Co-App/ SC/Mentor | IR | P | Population health | U of C | Yes | AB | No |
| Jones, Nina | Co-App/SC/Mentor | IR | P | Biomed | UofGuelph | No | ON | No |
| Ahmadi, Morteza | Co-App/Mentor | KU | Pr | Natural sciences | Qidni Labs | No | ON | No |
| Abrams, Mitch | Co-App/Mentor | Other^1^ | P | Cultural | McMaster | Yes | ON | No |
| Myles, Elizabeth | PAC/ Collab | KU | NP | N/A | KFoC | No | QC | Yes |
| Chun, Justin | PAC/ Trainee/Collab | ECR | P | Biomed | U of C | Yes | AB | Yes |
| Elliott, Meagan | PAC | IR | P | Clinical | U of C | Yes | AB | Yes |
| Lemarche, Caroline | PAC | ECR | P | Biomed | U of Montreal | Yes | QC | Yes |
| Young, Ann | PAC/ Trainee/ Collab | IR | P | Clinical | U of T | Yes | ON | Yes |
| Hemmelgarn, Brenda | PAC/ Institutional Rep/ Collaborator | KU, IR | P | Clinical, health systems, population health | U of A | Yes | AB | Yes |
| Woodlock, Tania | PAdC/Collab | KU | N/A | N/A | N/A | No | AB | No |
| MacPhee, Anne | PAdC/Collab | KU | N/A | N/A | N/A | No | NS | No |
| McCutcheon, Shanda | PAdC/Collab | KU | N/A | N/A | N/A | No | AB | No |
| Verdin, Nancey | PAdC/Collab | KU | N/A | N/A | N/A | No | AB | No |
| King, Andrew | Collab | KU | Pr | Biomed | Chinook Pharmaceuticals | No | BC | No |
| Harris, Heather | Collab | KU | NP | N/A | Can-SOLVE | No | BC | No |
| Clase, Catherine | Collab | IR | P | Clinical | CJKHD | yes | ON | No |
| Leblanc, Annie | Collab | IR | P | All | Laval | yes | QC | No |
| Gupta, Indra | Collab | IR | P | Biomed | McGill | yes | QC | No |
| Suri, Rita | Collab | IR | P | Clinical | McGill | yes | QC | No |
| Cordat, Emmanuelle | Collab | IR | P | Biomed | U of A | yes | AB | No |
| Hiremath, Swapnil | Collab | KU | P | Clinical | U of Ottawa | yes | ON | No |
| Jansen placeholder | Collab | KU | Pr | Biomed | Jansen Pharmaceutical | no | ON | No |
| Otsuka placeholder | Collab | KU | Pr | Biomed | Otsuka Pharmaceutical | no | ON | No |
| Samuel, Susan | Collab | IR | P | Health systems | UBC? | yes | AB | No |
| Ahmed, Sophia | Collab | IR | P | Clinical, health systems, population health | U of A | yes | AB | No |
| Sparks, Dwight | Collab | KU | NP | Clinical | CNTN | no | NL | No |
| Dionne, Janis | Collab | KU | P | N/A | CAPN | yes | BC | No |
| Mackay, Janice | Collab | KU | NP | N/A | CANNT | no | AB | No |
| Carr, Leah | Collab | KU | SP | N/A | NBHRF | no | NB | No |
| Marchand, Serge | Collab | KU | NP | N/A | FRQS | no | QC | No |

**NPA**, Nominated Principal Applicant; **PA**, Principal Applicant; **Co-App**, Co-applicant; **EDI**, Equity, Diversity, and Inclusivity Champion; **ECR**, Early Career Researcher; **SC**, Selection Committee; **PAC**, Platform Advisory Council; **PAdC**, Patient Advisory Committee; **IR**, Independent Researcher; **KU**, Knowledge User

*Sector includes public (P), private (Pr), semi-private, and non-profit

**Research discipline includes Biomedical, Clinical, Health System Services, Social, Cultural, Environmental and Population Health, Social Science, Humanities, Natural Science and Engineering.
