# Supplementary material for: The KRESCENT 2.0 Health Research Training Platform Application Process: Program Report
Source: Can J Kidney Health Dis. 2025 Aug 21;12:20543581251364309. doi: 10.1177/20543581251364309 (PMC12374036; doi:10.1177/20543581251364309)
Supplement: sj-docx-2-cjk-10.1177_20543581251364309 – Supplemental material for The KRESCENT 2.0 Health Research Training Platform Application Process: Program Report [file sj-docx-2-cjk-10.1177_20543581251364309.docx]

Supplementary Table 2: Detailed Gantt chart timeline.

| Step | Task | Deadline (2021) |
| --- | --- | --- |
| 1 | Registration |  |
| T01 | Identify team | 2-Feb |
|  | Identify NPA, PAs, mentors, other co-applicants |  |
|  | Create matrix |  |
|  | Identify potential researchers |  |
|  | Complete matrix information for all researchers |  |
|  | Fill gaps in matrix |  |
|  | Make decision on team |  |
|  | Confirm willingness of identified to participate |  |
|  | Finalize matrix |  |
| T02 | Review evaluation report for CIHR STIHR program | 15-Feb |
| T03 | Brainstorm uniqueness of KRESCENT 2.0 | 10-Mar |
|  | Gather mentor information and documents for registration | 25-Mar |
| T04 | Grant registration | 10-Apr |
|  | Submit registration CVs (NPAs and PAs) | 10-Apr |
| 2 | Internal application in full |  |
| T06 | Identify participants | 10-Apr |
|  | List all participants in 'Identify' task |  |
|  | List all participants in participant table |  |
|  | Submit CCV for all participants (except collaborators) |  |
| T07 | Write overview, title, lay title, lay abstract | 10-Apr |
| T08 | [Create descriptors](https://ucb40f15144f0a5de3b6efdc80aa.previews.dropboxusercontent.com/p/xls_html/ACX8HeVQiU-1IaNZHAY0Z7xGmcWQI8bHrrXws2OVp8blAOEVc4LmgJJA07DKbWhA9nwywONzxoTE3YJ3fIcj2UciXjj7-aovAWVTtM6IECl8LN4OQBOC852H-L2RuibzhPb7FAPlTpgzD9I0K8hLp1XP059Wjg6zYL44f79ONhlRpY5rzfP0MFiQ_LQRTlU8FkQihzN3zyj_v6qn5lqVXiTwJLGZt8Tk_LQH4Yd5FPhZM7kU2DG6kr7jvO7_TLLxxL4bN0NuLqqFkoCS6PU2Dl9lZ4E0jnzBixdComMJRQxJu6nEiqsiVlbEQGjzWc7Q6eQ/p.html) | 10-Apr |
| T09 | Write research proposal (20 pages) | 10-Apr |
| T10 | Complete proposal appendix | 10-Apr |
|  | Include bibliography and pre-existing training resources |  |
|  | Submit full CIHR academic CV | 30-Apr |
| T11 | Complete proposal summary (1 page) | 5-May |
| T12 | Completion of 1st draft | 7-May |
| 3 | Final application |  |
| T13 | Internal draft feedback |  |
| T14 | Create budget |  |
|  | ID institution to administer funds |  |
| T15 | Attach other application materials |  |
|  | Letters of support (NPA, 2 PA, mentors) |  |
|  | Collaboration letters (heads of partnering institutions) |  |
|  | EDI champion statement |  |
|  | Participant table |  |
|  | Organizational chart |  |
| T16 | Apply to 1 priority announcement |  |
| T17 | Complete internal review |  |
| T18 | Complete external review |  |
| T19 | Send grant to external editor |  |
| T20 | Provide list of potential reviewers |  |
| T21 | Gather/print/upload signature pages |  |
| T22 | Submit grant (internal review) | 14-Jun |
|  | Submit grant (external review) |  |
|  | Grant deadline | 21-Jun |
